# Identification of anoikis-related molecular patterns to define tumor microenvironment and predict immunotherapy response and prognosis in soft-tissue sarcoma

**DOI:** 10.3389/fphar.2023.1136184

**Published:** 2023-03-01

**Authors:** Lin Qi, Fangyue Chen, Lu Wang, Zhimin Yang, Wenchao Zhang, Zhi-Hong Li

**Affiliations:** ^1^ Department of Orthopedics, The Second Xiangya Hospital, Central South University, Changsha, China; ^2^ Hunan Key Laboratory of Tumor Models and Individualized Medicine, The Second Xiangya Hospital, Changsha, China; ^3^ Department of General Surgery, Changhai Hospital, Navy Military Medical University, Shanghai, China; ^4^ Department of Microbiology, Immunology & Molecular Genetics, University of Texas Long School of Medicine, UT Health Science Center, San Antonio, TX, United States

**Keywords:** soft-tissue sarcoma, anoikis, immune cell infiltration, tumor microenvironment, scoring system

## Abstract

**Background:** Soft-tissue sarcoma (STS) is a massive threat to human health due to its high morbidity and malignancy. STS also represents more than 100 histologic and molecular subtypes, with different prognosis. There is growing evidence that anoikis play a key role in the proliferation and invasion of tumors. However, the effects of anoikis in the immune landscape and the prognosis of STS remain unclear.

**Methods:** We analyzed the genomic and transcriptomic profiling of 34 anoikis-related genes (ARGs) in patient cohort of pan-cancer and STS from The Cancer Genome Atlas (TCGA) database. Single-cell transcriptome was used to disclose the expression patterns of ARGs in specific cell types. Gene expression was further validated by real-time PCR and our own sequencing data. We established the Anoikis cluster and Anoikis subtypes by using unsupervised consensus clustering analysis. An anoikis scoring system was further built based on the differentially expressed genes (DEGs) between Anoikis clusters. The clinical and biological characteristics of different groups were evaluated.

**Results:** The expressions of most ARGs were significantly different between STS and normal tissues. We found some common ARGs profiles across the pan-cancers. Network of 34 ARGs demonstrated the regulatory pattern and the association with immune cell infiltration. Patients from different Anoikis clusters or Anoikis subtypes displayed distinct clinical and biological characteristics. The scoring system was efficient in prediction of prognosis and immune cell infiltration. In addition, the scoring system could be used to predict immunotherapy response.

**Conclusion:** Overall, our study thoroughly depicted the anoikis-related molecular and biological profiling and interactions of ARGs in STS. The Anoikis score model could guide the individualized management.

## Introduction

Soft-tissue sarcoma (STS) is rare and accounts for approximate 1% of all adult malignancies (Gamboa et al., 2020), most commonly occurring in the extremities. In 2022, 13,190 people were newly diagnosed with STS and 5,130 people died of STS in United States (Siegel et al., 2022). STS was known as its heterogeneity which includes at least 100 different histologic and molecular subtypes. Genomic study has indicated that STS was mainly characterized by copy number variations but low mutation loads (Cancer Genome Atlas Research Network. Electronic address and Cancer Genome Atlas Research Network, 2017). However, a few genes (TP53, ATRX, RB1) showed highly recurrent mutations across different sarcoma types. These findings highlighted the importance of genetic alterations in STS, corresponding to its heterogeneity. Meanwhile, transcriptomic profiling of STS enhanced our understanding of STS biology and provided potential therapeutic targets. Transcriptomics can identify patients among different histological subtypes (Nielsen et al., 2002; Linn et al., 2003). Expression of some gene signatures were associated with prognosis of STS, such as hypoxia-inducible factor alpha (HIFA) and its targets (Francis et al., 2007).

In recent years, multiple molecular processes have been introduced to cancer biology and treatment such as the anoikis. Anoikis is a programmed cell death manner, happening when cells detached from appropriate extracellular matrix, which is a crucial mechanism in maintenance of plastic cell growth and attachment (Taddei et al., 2012). Cancer cells are characterized by insensitivity to anoikis since its survival and proliferation do not rely on adhesion to extracellular matrix (Cai et al., 2015). Thus, cancers represent a feature of anoikis resistance. In this scenario, figuring out the anoikis regulators of cancers contributes to the discovery of novel therapeutics, especially for cancer metastasis (Sakamoto and Kyprianou, 2010). For instance, in LKB1-deficient lung cancer, the PLAG1-GDH1 axis was reported to accelerate anoikis resistance through the CamKK2-AMPK pathway (Jin et al., 2018). Nuclear MYH9-induced CTNNB1 expression could facilitate gastric cancer cell anoikis resistance and induce metastasis. Similarly, it was reported that anoikis resistance in gastric cancer was regulated by TCF7L2 through transcriptionally activating PLAUR (Zhang et al., 2022), resulting in enhancement of metastasis. IQGAP1, a scaffolding protein that regulates cellular motility and extracellular signals, also reported to modulate the anoikis resistance and metastasis of hepatocellular carcinoma by accumulation of Rac1-dependent ROS and activation of Src/FAK signaling (Mo et al., 2021). These researches highlighted the critical role of anoikis profiling in various cancers.

Specifically, anoikis resistance also participate in the biology of STS. Recently, a study has conducted proteomic screens to identify suppressors of anoikis in Ewing sarcoma. The result indicated that the upregulation of IL1 receptor accessory protein (IL1RAP) significantly suppressed anoikis, which could be a new cell-surface target in Ewing sarcoma (Zhang et al., 2021). In a previous study, E-cadherin cell-cell adhesion was demonstrated to mediate suppression of anoikis by activating the ErbB4 tyrosine kinase in Ewing sarcoma (Kang et al., 2007).

Together, these findings have depicted a potential but limited role of anoikis in STS. More comprehensive studies are required to reveal the muti-omic profiling, regulator networks, microenvironments, targetable molecules, and prognostic predictors for STS. Further genotyping based on anoikis-related genes would help to understand the heterogeneity of STS, which is important to the personalized medicine. Therefore, in this study, we comprehensively analyzed the cross-talk of the anoikis-related genes (ARGs) and their molecular profiling in STS. We also focused on the impact of ARGs on tumor microenvironment, especially on the immune cell infiltration. Meanwhile, the stratification system and prognostic scoring model were established based on ARGs to guide the therapeutics for STS.

## Materials and methods

### Data collection and processing

The gene expression matrices of STS were downloaded from UCSC Xena (https://xenabrowser.net/) and GEO database (https://www.ncbi.nlm.nih.gov/geo/). Normal adipose and muscle tissue sample from Genotype-Tissue Expression (GTEx) database were used as normal control (https://gtexportal.org/home/). UCSC Xena has co-analyzed the TCGA data and GTEx data using UCSC bioinformatic pipeline (TOIL RNA-seq) for gene expression comparison. The copy number variations (CNVs), somatic mutation, and clinical information were downloaded from TCGA-SARC cohort. For pan-cancer analysis, data was derived from the TARGET Pan-Cancer (PANCAN) cohort. In GEO database, we identified two cohort of STS (GSE17674 and GSE63157) with prognosis data and one dataset of single-cell RNA-seq for STS (GSE131309). Moreover, we introduced a cohort of immunotherapy, in which the patients were treated with the combination of anti-PD-1 and anti-CTLA-4 therapy (Gide et al., 2019). By using this cohort, we analyzed the association between immunotherapy response and Anoikis score.

### Unsupervised clustering of ARGs

We identified the ARGs from GOBP_ANOIKIS term of Gene Set Variation Analysis (GSVA) database (http://www.gsea-msigdb.org/gsea/msigdb/cards/GOBP_ANOIKIS). Chromosome location of ARGs was plotted by the package “Rcircos” (version 1.2.1). Next, we conducted unsupervised clustering analysis using the 34 ARGs to define distinct clusters of patients. We set the key parameters of maxK = 9 and repetitions = 1,000 for algorithm packaged in “ConsensusClusterPlus” (Wilkerson and Hayes, 2010). Further, we recognized the differentially expressed genes (DEGs) (log2|FC|≥3, adjp <0.05) between Anoikis clusters by using the R package “limma” (version 3.48.3). Univariate COX regression analysis was utilized to recognize DEGs with significant prognostic relevance in STS.

As the prognostic DEGs were identified, we further input them into unsupervised clustering analysis and stratified patients into different Anoikis subtypes. These subtypes were more applicative and accurate since the DEGs reflected more comprehensive and common gene profiling.

### GSVA and Gene Ontology (GO) annotation

For the above defined clusters or subtypes, GSVA analysis was conducted to probe their biological characteristics by using the R package “GSVA” (version 1.40.1) (Hanzelmann et al., 2013). Meanwhile, biological differences between subgroups with high and low Anoikis score were also analyzed by GSVA. The h.all.v7.5.1 and c2.cp.kegg.v7.4 gene sets were downloaded from the Molecular Signatures Database (MSigDB). The R package “limma” (version 3.48.3) was utilized when comparing the differential expressed hallmark gene sets and tested using moderated t-statistics. The results were plotted using the R package “ggplot2” (version 3.3.5). Additionally, the R package “clusterProfiler” (version 4.0.5) was adopted for GO annotation. The significant enrichment was determined by false discovery rate (FDR) < 0.05.

### Evaluation of tumor immune infiltration

To assess the immune cell infiltration in tumor microenvironment, we applied the single-sample gene set enrichment analysis (ssGSEA), the marker genes of multiple types of immune cells were downloaded from previous study (Bindea et al., 2013). Infiltration level was normalized ranging from 0 to 1. Tumor mutation burden (TMB) signatures from published data (Mariathasan et al., 2018) were utilized to estimate the association between tumor microenvironment and biological processes. Moreover, we extracted signatures related to immunotherapy-predicted pathways and cancer-immunity cycles as previously reported (Chen and Mellman, 2013; Hu et al., 2021). Specifically, the cancer-immunity cycles containing seven steps: step one and two: cancer antigen release and presentation, step three: T-cell priming and activation, step four: immune cell recruitment, step five: immune cell infiltration into tumors, step six: T-cell recognition of cancers, step seven: killing of cancer cells. These cycles were applied to guide frameworks for immunotherapy. We used GSVA to calculate the signatures scores of immunotherapy-predicted pathway and cancer-immunity cycles as previously described. The associations between Anoikis score and GSVA scores of different gene sets were compared by using the R package “ggcor” (version 0.9.4.3).

### Establishment of the anoikis scoring model

In order to applied the above findings in more patients, we next generated the anoikis scoring system based on our previous established Anoikis clusters. DEGs between Anoikis cluster C1 and C2 were identified and Univariate COX regression analysis was conducted to recognize prognosis relevant DEGs. The prognostic DEGs were then analyzed using principal component analysis (PCA) and calculated for signature scores. This method was advantageous in identification of the score of the set with most significant correlation and elimination of unrelated blocks. To calculate the Anoikis score, the formula of *Σ*(*PC1*_*i*_ *+ PC2*_*i*_) was applied where *i* was the expression of the enrolled prognostic DEGs. On this basis, patients were divided into the high and low Anoikis score group according to a cut-off value determined by the algorithm.

### Single-cell transcriptome analysis

In this study, we used a single-cell RNA-seq dataset (GSE131309) from published study (Jerby-Arnon et al., 2021). The data were analyzed following standard pipeline of the package “Seurat” (version 4.0.5). Gene expression was normalized by LogNormalize (scale factor = 10,000). 2,000 highly variable genes (HVGs) were then recognized within the function of FindVariableGenes. 25 PC were picked up based on the result of ElbowPlot. Subsequently, we performed the cell clustering and t-distributed stochastic neighbor embedding (t-SNE) to figure out the cell subpopulations. The same labels from the data resource were used for specific cell cluster annotation, as described in previous study (Jerby-Arnon et al., 2021). Expression of specific genes was illustrated in t-SNE plots.

### Prediction of chemotherapeutic sensitivity

Drug response data were retrieved from the Genomics of Drug Sensitivity in Cancer (GDSC) (https://www.cancerrxgene.org/downloads/anova). The GDSC database provides the drug sensitivity data and genetic correlation for more than 1,000 genetically characterized human cell lines (Yang et al., 2013). Drug response data of 518 compounds targeting 24 pathways were identified. IC50 and drug sensitivity score were utilized to assess the chemotherapeutic sensitivity, as calculated by the R packages “pRRophetic” (version 0.5) and “oncoPredict” (version 0.2) (Iorio et al., 2016; Maeser et al., 2021).

### Cell lines and real-time PCR

The human synovial sarcoma (SW-982) and liposarcoma cell line (SW-872) were purchased from the Procell Life Science & Technology Co., Ltd. Primary human skin fibroblast cell line (HSF) was acquired from Fenghui Biotechnology Co., Ltd. The primary hSS-005R cell line was established by our laboratory. They were cultured in Dulbecco’s modified Eagle medium (DMEM) completed with 10% fetal bovine serum (FBS) and 1% Penicillin-Streptomycin at 37 °C and 5% CO_2_.

For real-time PCR analysis of mRNA expression, 2×10^5^ cells were cultured in six well plates for 24 h and the RNA Express Total RNA Kit (M050, NCM Biotech, China) was used for subsequent total RNA extraction. RevertAid First Strand cDNA Synthesis kit (K1622, Thermo Fisher Scientific, United States) was utilized for cDNA synthesis. For each sample, 50 ng cDNA was mixed with Hieff^®^ qPCR SYBR Green Master Mix (11201ES03, YEASEN, China) and gene specific primers following the manufacturer’s protocol. Reactions were performed on the Applied Biosystems QuantStudio (Thermo Fisher Laboratories). Real-time PCR experiments were repeated using three biological replicates. The primer sequences were as follow: GAPDH, 5′- CAG​GAG​GCA​TTG​CTG​ATG​AT -3' (forward), 5′- GAA​GGC​TGG​GGC​TCA​TTT-3' (reverse); E2F1, 5′- ACG​TGA​CGT​GTC​AGG​ACC​T -3' (forward), 5′- GAT​CGG​GCC​TTG​TTT​GCT​CTT -3' (reverse); SNAI2, 5′- TGT​GAC​AAG​GAA​TAT​GTG​AGC​C -3' (forward), 5′- TGA​GCC​CTC​AGA​TTT​GAC​CTG -3' (reverse); DAPK2, 5′- GGG​ACG​CCG​GAA​TTT​GTT​G -3' (forward), 5′- TTC​CTG​CTT​CGT​GTC​TCC​CA -3' (reverse).

### Full-length transcriptome analysis

We performed full-length mRNA-seq on four STS samples and four paired normal tissues (GSE198568). Total RNA was extracted from fresh frozen samples for full-length transcriptome analysis. The sequencing was performed by Biomarker Technologies (Biomarker Technologies Ltd., Beijing, China) following the operation protocols of Oxford Nanopore Technologies (Oxford Nanopore Technologies, Oxford, United Kingdom). Data were analyzed in accordance with the pipeline provided by Biomarker Technologies Ltd.

### Statistical analysis

R software (version 4.1.0) was used for statistical analysis. We conducted the spearman correlation test when calculating the correlations of ARGs. Student’s t-tests and Wilcoxon signed-rank test were conducted for parametric comparisons and non-parametric comparisons. Multiple groups comparisons were tested by one-way ANOVA or Kruskal–Wallis test. Log-rank test was applied in survival analysis. The prognostic factors were determined by Univariate and multivariate Cox regression. To assess the accuracy of model, Receiver operating characteristic (ROC) curves were plotted and area under the curve (AUC) was calculated by using R package “timeROC” (version 0.4). The optimal cut-off value of Anoikis scores was determined by using the package “survminer” (version 0.4.9). Besides, chi-square or Fisher exact tests was adopted to compare clinical characteristics in different groups. *p*-value <0.05 was defined as statistical significance.

## Results

### Pan-cancer analysis of ARGs

**FIGURE 1:**
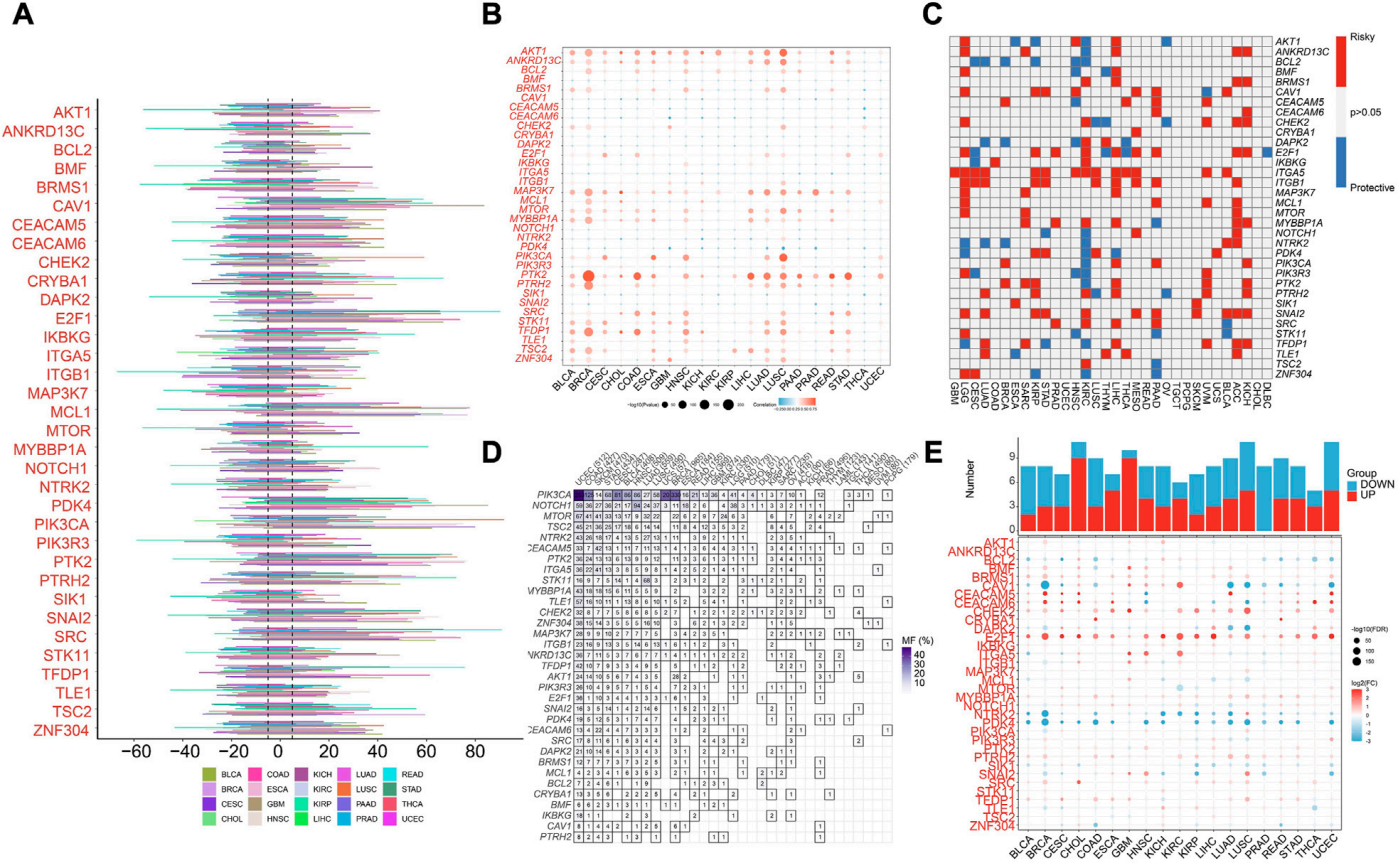
Pan-cancer analysis of Anoikis-related genes (ARGs) in pan-cancer TCGA data. **(A)** The illustration of somatic copy number variance (SCNV) of ARGs in different cancer types. The percentage of amplification and deletion was annotated. **(B)** The correlation of SCNV and expression of ARGs within different cancer types. **(C)** The prognostic effects of the expression of ARGs across different cancer types. Red indicates the risk factor, and blue indicated the protective factor. **(D)** The mutation frequency of ARGs in different cancer types. **(E)** The expression patterns of ARGs between tumor and normal samples in different cancer types. The upper histograms illustrate the number of significantly differentially upregulated (red) and downregulated (blue) genes.

We first analyzed the profiling of ARGs in pan-cancer level. Copy number variance (CNV) analysis of ARGs indicated CNV gain of CVA1, E2F1, MCL1, PDK4, PIK3CA, PTK2, SNAI2, and SRC in various cancer types (Figure 1A). Significant correlation between SCNV and expression of PTK2 was found in different cancer types (Figure 1B). As reveled by survival analysis, high expression of most ARGs suggested high risk effect for LGG, LIHC, ACC and KICH but protective effect for KIRC (Figure 1C). Besides, ITGA5 and ITGB1 were risk factors for multiple cancer types (Figure 1C). Among the 34 ARGs, PIK3CA showed the highest mutation frequency in different cancer types (Figure 1D). E2F1 and CHEK2 were highly expressed across most cancer types compared to normal samples, while PDK4 and NTRK2 were decreased in various cancers (Figure 1E).

### Genomic and transcriptional landscapes of ARGs in STS

**FIGURE 2:**
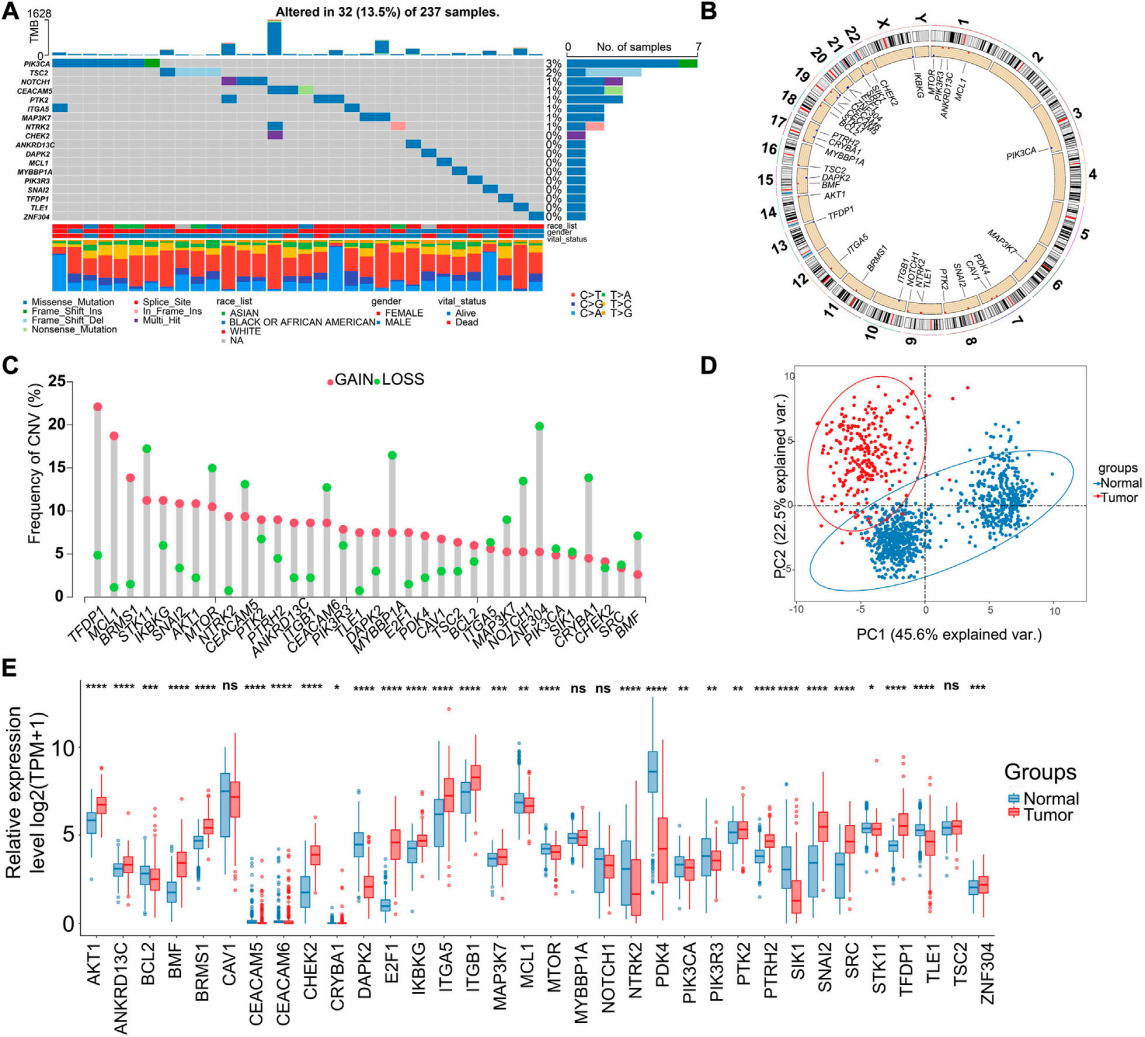
Genomic and transcriptional landscapes of ARGs in soft-tissue sarcoma (STS) in TCGA database. **(A)** The mutation frequency of ARGs (Top 18) in 237 patients with STS in TCGA database. **(B)** The specific location of ARGs on the human chromosomes. **(C)** The SCNV of ARGs in patients with STS in TCGA database. Red indicates CNV gain, and green indicates CNV loss. **(D)** The principal component analysis (PCA) of ARGs expression to identify tumor among normal samples based on the TCGA-GTEx database. Red indicates tumor samples, blue indicate normal tissues. **(E)** The expression of ARGs between tumor (red) and normal samples (blue) based on the TCGA-GTEx database. *, 0.01 ≤ *p* < 0.05; **, 0.001 ≤ *p* < 0.01; ***, 0.0001 ≤ *p* < 0.001; ****, *p* < 0.0001; ns, *p* ≥ 0.05.

**FIGURE 3:**
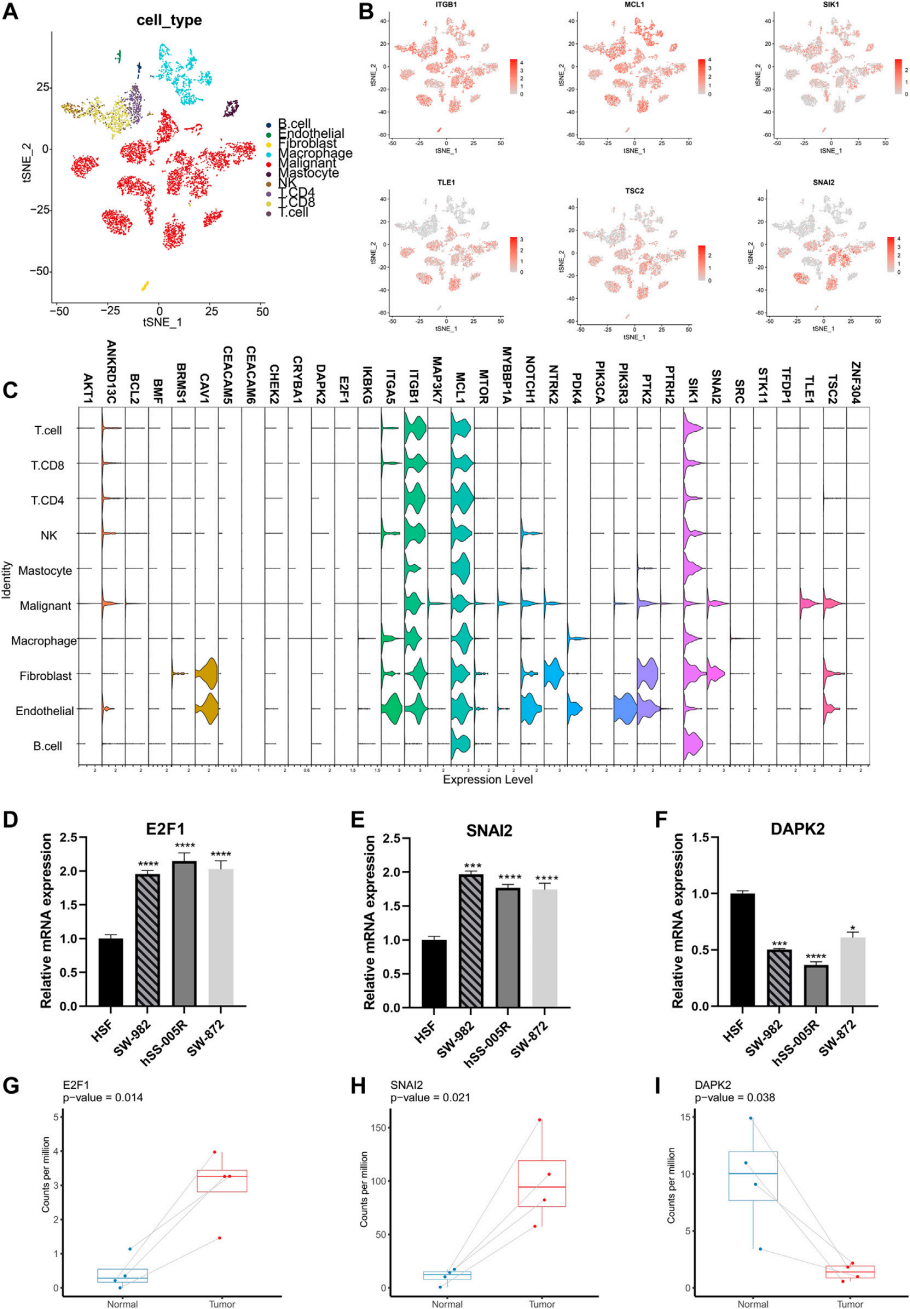
Validation of expression patterns of ARGs at single-cell resolution. **(A)** The t-distributed stochastic neighbor embedding (t-SNE) plot showing specific cell types of STS. **(B)** The t-SNE plots showing the expression of ARGs in different cell types. **(C)** The violin plots illustrating expression levels of ARGs across different cell types. **(D–F)** Validation of expression of ARGs between STS cell lines and the control cell line by the real-time PCR. Real-time PCR experiments were repeated using three biological replicates. **(G–I)** The box plots illustrating the expression of ARGs between STS and matched adjacent normal tissues based on our own sequencing data. *, 0.01 ≤ *p* < 0.05; ***, 0.0001 ≤ *p* < 0.001; ****, *p* < 0.0001.

More specifically, the ARGs were analyzed in STS cohort. Only 32 (13.5%) of 237 samples showed ARGs-related mutations, concentrating within 18 ARGs (Figure 2A). Most ARGs located in chromosome 1, 9, 17, 19 (Figure 2B). The SCNV frequency of ARGs were depicted in Figure 2C. Notably, the expression profiling of 34 ARGs could discriminate against tumor and normal tissues (Figure 2D) since most of them showed significant differential expression (Figure 2E). In order to specialize the expression pattern of ARGs, we next visualized their expression in single cell transcriptomics from GSE131309 (Figures 3A, B). We noticed that ITGB1, MCL1, and SIK1 broadly expressed in all cell types while TLE1, TSC2, and SNAI2 were mainly clustered in malignant subtypes (Figures 3B, C; Supplementary Figure S1). As validated by real-time PCR, the expression of E2F1 and SNAI2 were significantly higher in STS cell lines including SW-982, hss-005R, and SW-872 compared to HSF cell line, while DAPK2 was lower in STS cell lines (Figures 3D–F). Additionally, the consistent results were identified in our own sequencing data of four pairs of STS and normal samples (Figures 3G–I).

### Cross-talk of ARGs and identification of anoikis clusters

**FIGURE 4:**
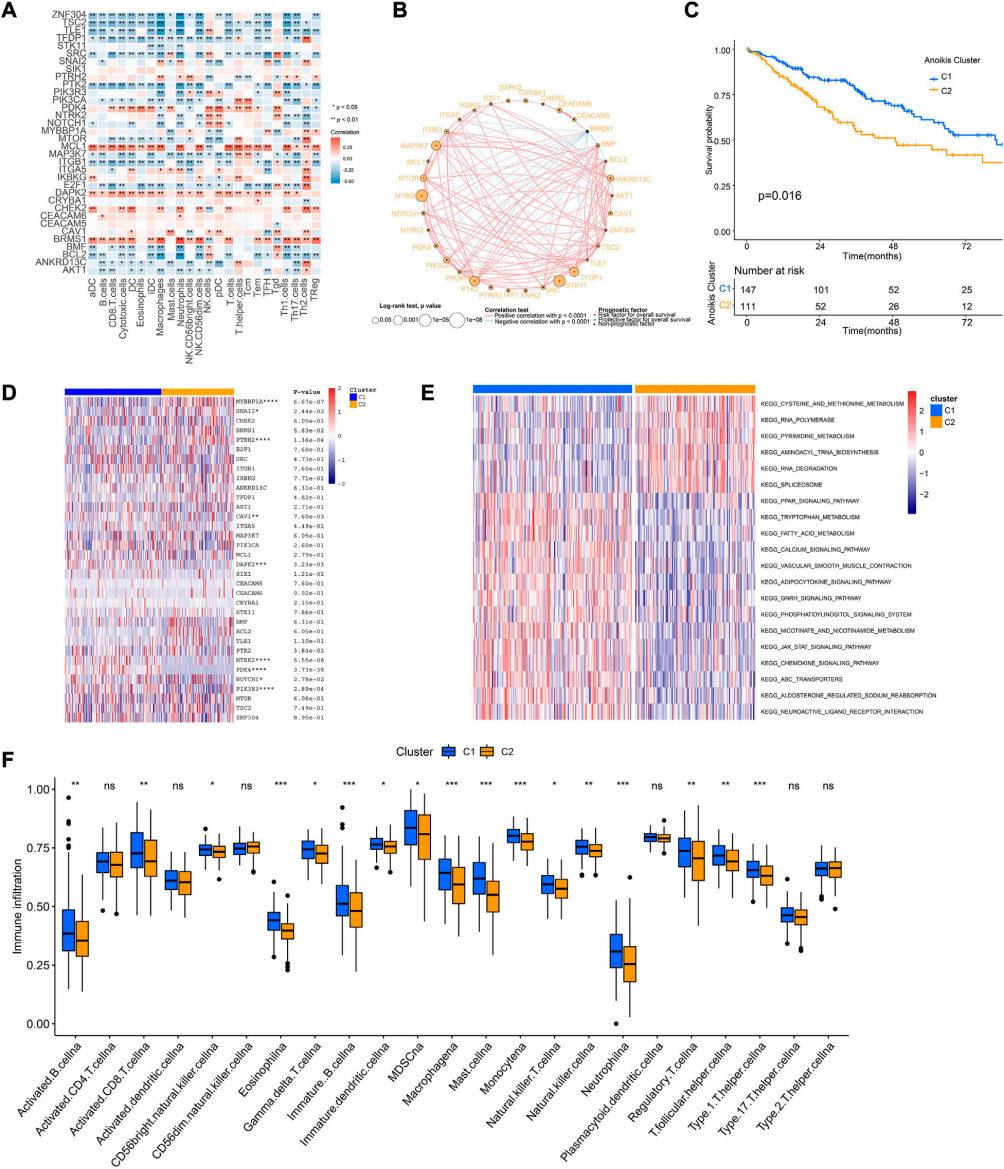
Cross-talk of ARGs and identification of Anoikis clusters. **(A)** The correlation analysis of the expression of ARGs and signatures of immune cells. Red indicated positively associated and blue indicated negatively associated. **(B)** The correlation network of ARGs in the TCGA-SARC cohort. The significance of the prognostic effects was illustrated by the circle size. **(C)** The Kaplan-Meier curve comparing the survival between different Anoikis clusters. **(D)** The heatmap of ARGs between different Anoikis clusters. **(E)**The gene set variation analysis (GSVA) illustrating pathways significantly enriched between different Anoikis clusters. **(F)** The infiltrations of different immune cells between different Anoikis clusters. *, 0.01 ≤ *p* < 0.05; **, 0.001 ≤ *p* < 0.01; ***, 0.0001 ≤ *p* < 0.001; ****, *p* < 0.0001; ns, *p* ≥ 0.05.

Tumor immune microenvironment is a key regulator of tumor progression, in which the immune cells cross-talk with other cell types and impact their predestination. Through correlation analysis of the expression pattern of ARGs and signatures of immune cells, we found that expression of MCL1, DAPK2, PDK4, and BRMS1 were positively correlated with most immune cells (Figure 4A). The network of 34 ARGs displayed a comprehensive landscape of the interactions (Figure 4B). Among them, most ARGs such as BMF, BCL2, ANKRD13C, AKT1, ZNF304, TSC2 showed positive correlation with other genes, but BRMS1 negatively correlated with most ARGs (Figure 4B). These findings indicated the interactive patterns of ARGs.

Further, we conducted unsupervised consensus clustering to identify distinct expression patterns of ARGs in different patients (Supplementary Figure S2). Consequently, 258 patients were clustered into two clusters by using K = 2 as the optimal index based on elbow method (Krolak-Schwedt and Eckes, 1992), named as C1 and C2 containing 147 and 111 patients respectively. The two clusters showed distinct prognosis (*p* = 0.016), ARGs expression patterns, and pathway enrichment patterns (Figures 4C–E), indicating the different characteristics between them. Specifically, patients of cluster C1 showed better survival and improved immune infiltration patterns (Figures 4C, F). GSVA showed that Cluster C1 were positively enriched in chemokine signaling and JAK-STAT signaling pathways (Figure 4E).

### Identification of distinct anoikis subtypes and related biological characteristics

**FIGURE 5:**
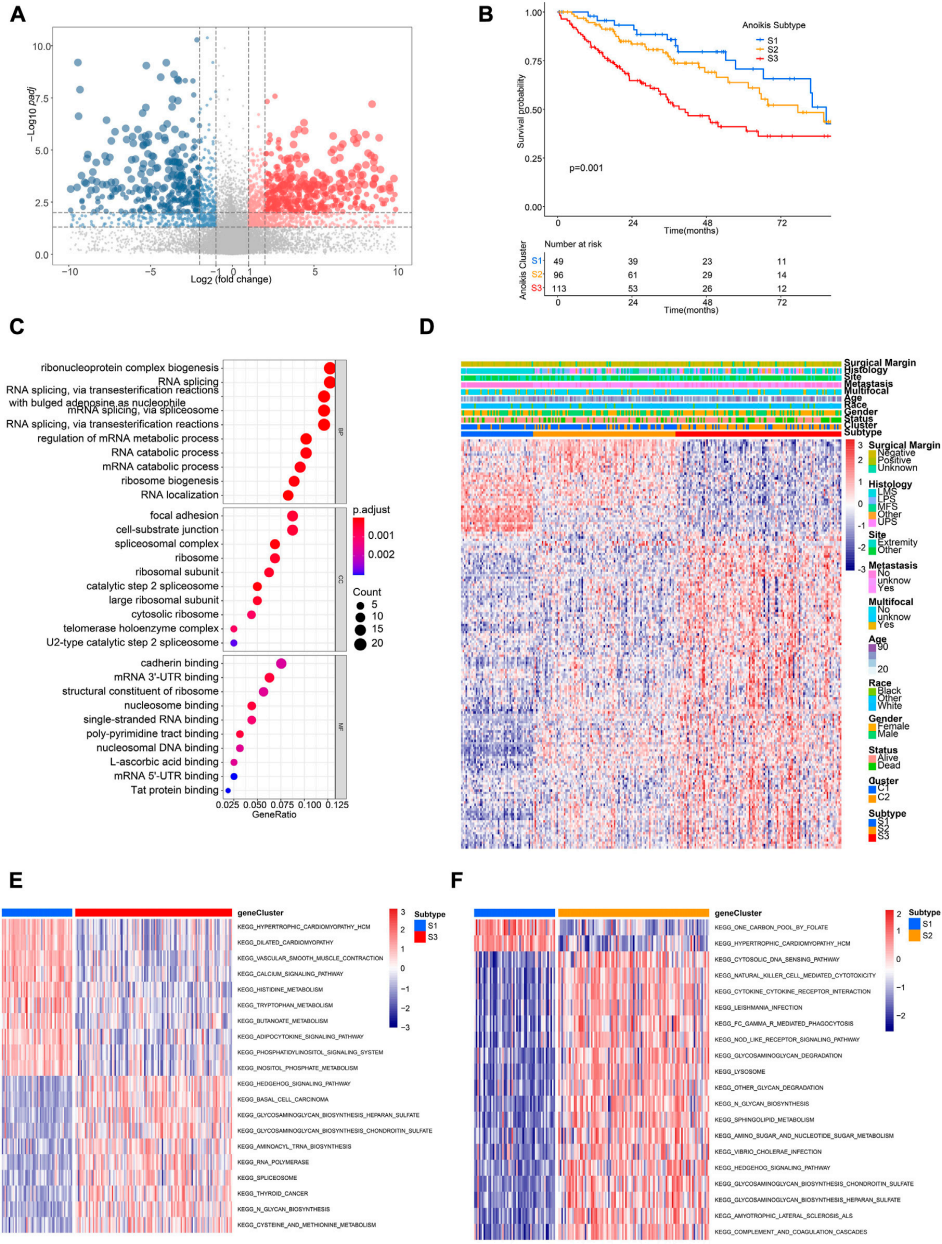
Identification of distinct Anoikis subtypes and related biological characteristics. **(A)** The volcano plot showing significantly differentially expressed genes (DEGs) between different Anoikis clusters (C2 versus C1). Genes significantly upregulated were marked in red, while genes significantly downregulated were marked in blue. **(B)** The Kaplan-Meier curve comparing the survival between different Anoikis subtypes. **(C)** Gene Ontology (GO) enrichment analysis of DEGs identified in the above resulted. BP, biological process; CC, cellular component; MF, molecular function. **(D)** The unsupervised clustering of TCGA-SARC cohort based on the ARGs-related DEGs. **(E, F)** The GSVA comparing pathways significantly enriched among distinct Anoikis subtypes.

In order to further identify distinct patient groups based on the characteristic of Anoikis clusters, we performed unsupervised consensus clustering using DEGs between cluster C1 and C2 (Figure 5A; Supplementary Figures 3A–F). As a result, three subtypes (S1, S2, S3) were identified, with the patient number of 49, 96, 113 respectively. Patients of the three subtypes were significantly different in survival (Figure 5B). Besides, the DEGs were enriched in GO terms of ribonucleoprotein complex biogenesis, RNA splicing, focal adhesion, cell-subtract junction, cadherin binding, etc. (Figure 5C). Gene expression patterns of three subtypes were distinct but the clinical characteristics were irregular (Figure 5D). Pathway analysis of different subtypes were conducted to identify corresponding characteristics. GSVA suggested the enrichment of hedgehog signaling, basal cell carcinoma, and glycosaminoglycan biogenesis in S3 subtype (Figure 5E), while the pathways of cytosolic DNA sensing, natural killing cell mediated cytotoxicity, and cytokine-cytokine receptor interaction were enriched in S2 subtype (Figure 5F). Interestingly, subtype S2 showed higher infiltration of most immune cells compared to S1 and S2 (Supplementary Figure S3G).

### Establishment and validation of anoikis score

**FIGURE 6:**
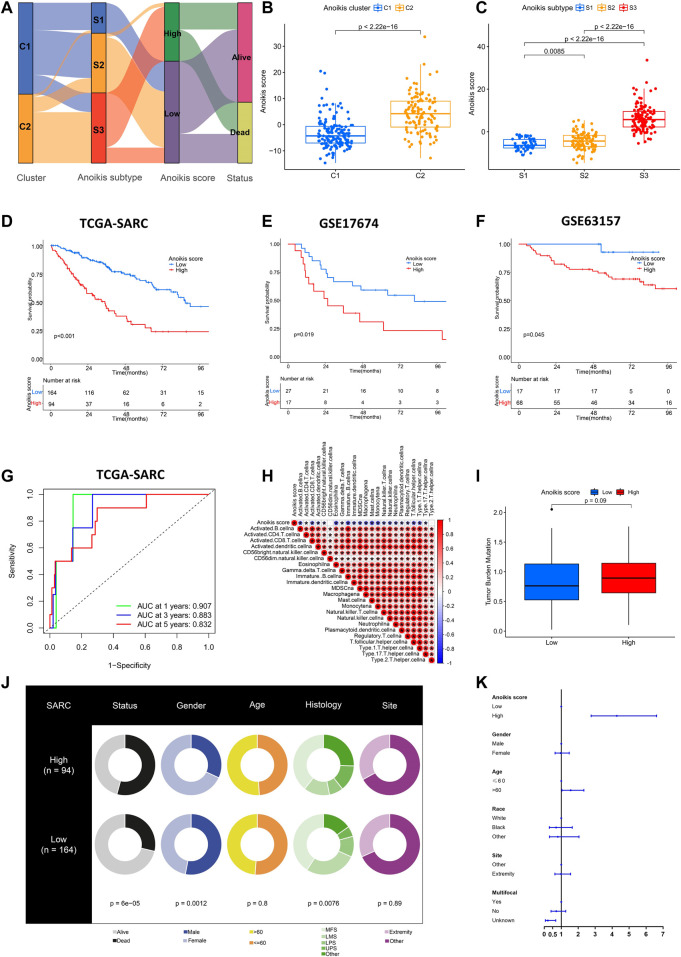
Establishment and validation of Anoikis score. **(A)** Alluvial diagram showing the relations among Anoikis clusters, Anoikis subtypes and Anoikis score groups. **(B, C)** The box plots illustrating the Anoikis score in different Anoikis clusters and Anoikis subtypes. **(D–F)** The Kaplan-Meier curves comparing the survival between low (blue) and high (red) Anoikis score groups in TCGA-SARC cohort **(D)**, GSE17674 **(E)** and GSE63157 **(F)**. **(G)** The time-dependent receiver operating characteristic curve (ROC) assessing the predictive performance of Anoikis score in TCGA-SARC cohort. **(H)** The correlation analysis between Anoikis score and signatures of immune cells. Red indicated positively associated and blue indicated negatively associated. **(I)** The box plot of tumor mutation burden (TMB) between low and high Anoikis score groups in TCGA-SARC cohort. **(J)** The pie plots showing proportions of different clinical characteristics between low and high Anoikis score groups in TCGA-SARC cohort. **(K)** The forest plot illustration multi-variate Cox analysis including clinical information and Anoikis score. *, *p* < 0.05.

As displayed above, the identification of Anoikis clusters (C1, C2) and Anoikis subtypes (S1, S2, S3) helped to classify patients with different gene expression patterns. Nevertheless, they were limited within the TCGA-SARC cohort. Therefore, we further established the Anoikis score based on DEGs between Anoikis clusters C1 and C2 to apply this model in external cohorts. The flow diagram was illustrated in Figure 6A. The Anoikis score was significantly different among Anoikis clusters or Anoikis subtypes (Figures 6B, C). Patients were then divided into the high Anoikis score and low Anoikis score group by an algorithm calculated cut-off value. Patients with high Anoikis score showed poor prognosis in TCGA-SARC cohort (*p* < 0.001) (Figure 6D). External validation using GSE17674 (*p* = 0.019) and GSE63157 (*p* = 0.045) data further confirmed this result (Figures 6E, F). The AUC also suggested the reliability of Anoikis score in 1-, 3-, and 5-year survival prediction, with the values of 0.907, 0.883, and 0.832 respectively (Figure 6G). Notably, the Anoikis score was negatively correlated with multiple types of innate immune cells and adoptive immune cells including B cells, Macrophages, and various subtypes of T cells (Figure 6H), suggesting the potential of Anoikis score in STS immune infiltration prediction. There was a slight difference in TMB between high and low Anoikis score group (Figure 6I). Additionally, groups with high and low Anoikis score showed differences in clinical characteristics including survival status (*p* < 0.001), gender (*p* < 0.001), and histology (*p* < 0.001), but not in age and tumor site (Figure 6J). Multivariate Cox regression analysis indicated that high Anoikis score was a significant risk factor for STS (Figure 6K; Supplementary Figure S4). Together, these findings demonstrated the reliability of our Anoikis score model in prognostic prediction for STS.

### The genomic and transcriptional characteristics between anoikis score groups

**FIGURE 7:**
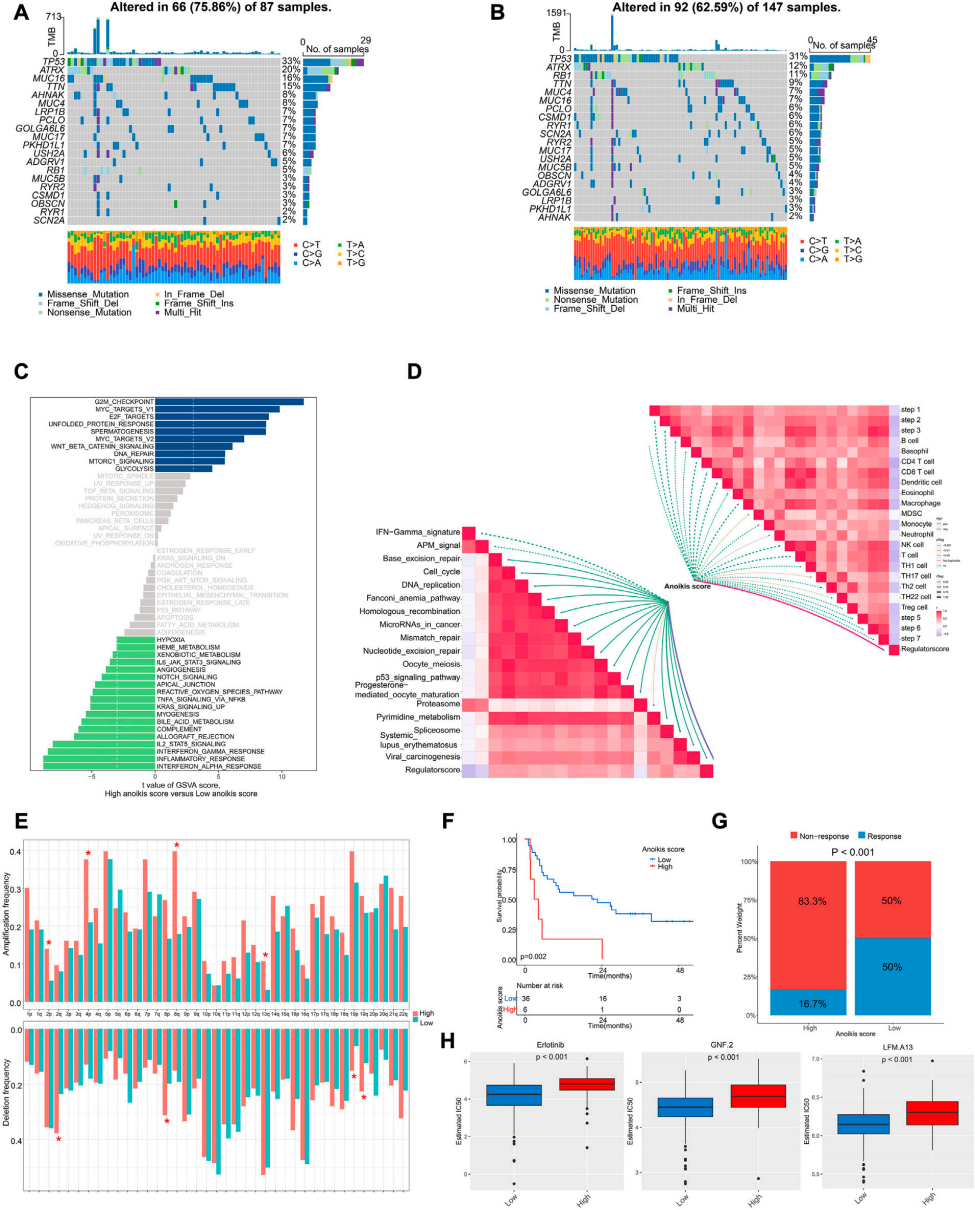
The genomic and transcriptional characteristics between Anoikis score groups. **(A, B)** The differences in mutation frequency between high **(A)** and low **(B)** Anoikis score groups. **(C)** The GSVA illustrating significantly differently enriched pathways between Anoikis score groups. **(D)** The correlation analysis of Anoikis score with immunotherapy-predicted pathways and cancer immunity cycles. **(E)** The frequency of arm-level amplification and deletion between Anoikis score groups. **(F)** The Kaplan-Meier curve comparing the survival between low and high Anoikis score groups in an immunotherapy cohort. **(G)** The rates of clinical response between Anoikis score groups in an immunotherapy cohort. **(H)** The box plots showing significant differences in the estimated IC50 of several drugs between Anoikis score groups in TCGA-SARC cohort. *, *p* < 0.05.

Next, we interrogated the genomic and transcriptional profiling between high and low Anoikis score groups. We observed a higher frequency of mutation in high Anoikis score group with alteration in 66 (75.86%) of 87 samples (Figure 7A), compared with low Anoikis score group with mutations in 92 (62.59%) of 147 samples (Figure 7B). Noteworthily, the frequency of arm-level amplification and deletion seems to be higher in high Anoikis score group compared to low group (Figure 7E). Considering the enriched pathways in different Anoikis score groups, we found positive enrichment of pathways including G2M checkpoint, MYC targets, and E2F targets in high Anoikis score group but negative enrichment of pathways including interferon alpha response, inflammation response, and interferon gamma response (Figure 7C). This result was consistent with previous finding (Figure 6H) that high Anoikis score indicated poor immune infiltration. Moreover, we analyzed the correlation of Anoikis score with immunotherapy-predicted pathways and cancer immunity cycles. As a result, the Anoikis score was significantly negative associated with various immune cells including B cell, CD4^+^ T cells, CD8^+^ T cells, dendritic cells, etc. Meanwhile, Anoikis score was positively correlated with most immunotherapy-predicted pathways such as Base excision repair, cell cycle, and DNA replication (Figure 7D).

Because of the close relationship of Anoikis score and immune status, we further analyzed the Anoikis score in an immunotherapy cohort. Interestingly, patients with high Anoikis score showed poor survival (*p* = 0.002) (Figure 7F) and poor response to immunotherapy (*p* < 0.001) (Figure 7G). Additionally, we utilized the GDSC database to screen for drugs with different response in high and low Anoikis score groups. Surprisingly, we identified three drugs with higher IC50 in high Anoikis score group compared to low score group, namely, erlotinib (*p* < 0.001), GNF.2 (*p* < 0.001) and LFM.A13 (*p* < 0.001) (Figure 7H). These findings could provide potential methods for individualized immunotherapy of STS patients.

## Discussion

STS is an uncommon and heterogeneous tumor with limited treatment currently (Linch et al., 2014). Several studies have explored the genomic and transcriptomic characteristics of STS to uncover the molecular profiling and find new therapeutic targets. Anoikis, a critical process of cell death, has shown great impact on STS biology, predominantly through a mechanism of anoikis resistance, which could create a microenvironment suitable for tumor metastasis (Kang et al., 2007; Zhang et al., 2021). Although the intriguing conclusions have been made, there is a lack of comprehensive analysis and applicable predictive model for ARGs in STS. The interactions between ARGs and tumor microenvironment, especially the immune cell infiltration, have not been recognized for STS. In the present study, we conducted comprehensive analysis of the 34 ARGs in STS.

In spite of the fact that all cancers are molecularly distinct, many of them share common driver mutations or characteristics of transcriptional regulation (Ciriello et al., 2013). We first analyzed the profiles of ARGs at pan-cancer level. Several ARGs showed gain of CNVs such as E2F1, MCL1, and PIK3CA across multiple cancers. CNVs of E2F1 were reported previously in various type of cancers to be associated with cancer susceptibility (Nelson et al., 2006; Rocca et al., 2017; Rocca et al., 2019; Rocca et al., 2021). MCL1 also displayed CNVs in non-small lung cancer and uterine cervix adenocarcinoma and impact on survival of patients (Yin et al., 2016; Lin et al., 2020). Similarly, PIK3CA acquired CNVs in a wide-range of cancers which regulated the cancer progression and prognosis (Yamamoto et al., 2008; Brauswetter et al., 2016; Migliaccio et al., 2022). Interestingly, PIK3CA showed the highest frequency of mutations among all ARGs in different cancers, which was consistent with previous studies (Mei et al., 2016; Mosele et al., 2020).

In STS, mutation frequency of PIK3CA was also at the top of ARGs list, indicating its critical role in STS biology. Despite this, the overall mutation burden of ARGs in STS was relatively low. The expression of most ARGs were differentially expressed so that the expression pattern could discriminate between STS and normal tissues. Differential expression of some ARGs was further confirmed by real-time PCR and our own sequencing data. For unbiased high-resolution snapshots of gene expression programs, single-cell RNA sequencing is the preferred method. Single-cell resolved gene expression profiles offer several key advantages over bulk population sequencing (Kanev et al., 2021). Notably, by single-cell transcriptomic analysis, we found that the expression of ARGs showed cell-type specificity, e.g., ITGB1, MCL1, and SIK1 highly expressed in multiple cell types while TLE1, TSC2, and SNAI2 were predominantly identified in malignant subtypes. This characteristic could help guiding the discovery of new therapeutic targets. Single-cell transcriptomics in prostate cancer revealed the high expression of MCL1 in persistent senescent tumor cells, a kind of metabolically active cell that promoted tumor proliferation and metastatic dissemination (Troiani et al., 2022). Hence, MCL1 maybe a potential indicator for cancer malignancy.

Next, we established the clustering system for STS based on 34 ARGs by using unsupervised consensus clustering. Two clusters were recognized (C1 and C2), in which the cluster C1 was characterized by better prognosis and improved immune cell infiltration. We speculated that the distinct ARGs patterns in cluster C1 resulted in a tumor microenvironment suitable for immune cell response. As expected, pathway analysis indicated the enrichment of chemokine signaling and JAK-STAT signaling in cluster C1. Increase of chemokine contributed to the improvement of immune cell engraftment, such as T cells (Dangaj et al., 2019). The IFNγ-JAK-STAT signaling was also a determinant for chemokine expression (Xu et al., 2019). To further classify patients based on Anoikis clusters, we performed unsupervised consensus clustering based on DEGs between C1 and C2. Subsequently, three Anoikis subtypes with different characteristics were established (S1, S2, S3). We noticed that S1 showed the best prognosis while S2 was characterized by optimal immune infiltration. Compared with S3, the S1 subtype was enriched in several metabolic pathways such as histidine metabolism, tryptophan metabolism, butanoate metabolism, and adipocytokine signaling pathway. Among them, the histidine metabolism was associated with good response of cancer therapy (Frezza, 2018). However, the tryptophan metabolism and adipocytokine signaling pathway could promote cancer progression in other cancers (Rose et al., 2004; Platten et al., 2019). This inconsistent conclusion may be explained by the heterogeneity in different cancer types, further studies are required for exploration of the metabolism-related mechanisms and the cancer suppression metabolic niche in specific STS subtype. Not surprisingly, we also observed the enrichment of cytokine-cytokine receptor interaction in S2. It was reported that higher level of TMB was associated with poorer in cancer patients, and the risk scores of STS patients with higher risk score were also higher in our study, which needs further research (Valero et al., 2021).

Moreover, we built an anoikis scoring system according to the prognostic DEGs between cluster C1 and C2. The anoikis scoring system could be utilized to calculate specific score of individual patients. The system was effective in prediction of prognosis in multiple cohort which was of great potential in clinical guidance. The group of low Anoikis score showed better prognosis and immune infiltration. Similarly, the low Anoikis score group was enriched in immune-related pathway such as IL6 JAK-STAT3 signaling, TNFA signaling, complement, INFγ response, INFα response, and inflammatory response. Further, the Anoikis score may also serve as an indicator for the response of immunotherapy. Similar findings were also reported in other cancer types, as ARGs were significantly associated with TME (Guizhen et al., 2022; Zhang et al., 2023). Although the anoikis scoring system achieved good predictive performance, high intratumor heterogeneity between samples may limit further application of this tool. Besides, larger sample size is needed to validate results in the future.

## Conclusion

Taken together, this study comprehensively analyzed the anoikis profiles in STS for the first time. We unraveled the profiling and interactions of ARGs in both the pan-cancer levels and STS, figuring out the critical role of ARGs in tumor biology. The establishment of Anoikis subtypes reflected the heterogeneity of ARGs between patients regarding the prognosis and immune cell infiltration. The Anoikis scoring system further provided individualized assessment for prognosis and immune response, which could guide personalized treatment for STS.

## Data Availability

Publicly available datasets were analyzed in this study. This data can be found here: UCSC Xena (https://xena.ucsc.edu/) and GEO database (https://www.ncbi.nlm.nih.gov/geo/) with accession No.GSE17674, GSE63157, GSE131309, GSE198568.

## Supplementary material

SUPPLEMENTARY FIGURE S1 The t-SNE plots of the expression of ARGs in different cell types.

SUPPLEMENTARY FIGURE S2 The identification of Anoikis clusters. **(A-E)** Consensus clustering based on ARGs (K = 2-6). **(F)** Consensus cumulative distribution function (CDF) Plot based on ARGs.

SUPPLEMENTARY FIGURE S3 The identification of Anoikis subtypes. **(A-E)** Consensus clustering based on ARGs-realted DEGs (K = 2-6). **(F)** Consensus cumulative distribution function (CDF) Plot based on ARGs-realted DEGs. **(G)** The infiltrations of different immune cells between different Anoikis subtypes. ***, 0.0001 ≤ p <0.001.

SUPPLEMENTARY FIGURE S4 Subgroup analysis of Anoikis score based on clinical characteristics. The Kaplan-Meier curves comparing the survival between low and high Anoikis score groups in different genders **(A-B)**, ages **(C-D)**, histology **(E-I)** and tumor sites **(J-K)**.
