# Supplementary figures and images for: Identification of anoikis-related molecular patterns to define tumor microenvironment and predict immunotherapy response and prognosis in soft-tissue sarcoma
Source: Front Pharmacol. 2023 Mar 1;14:1136184. doi: 10.3389/fphar.2023.1136184 (PMC10014785; doi:10.3389/fphar.2023.1136184)

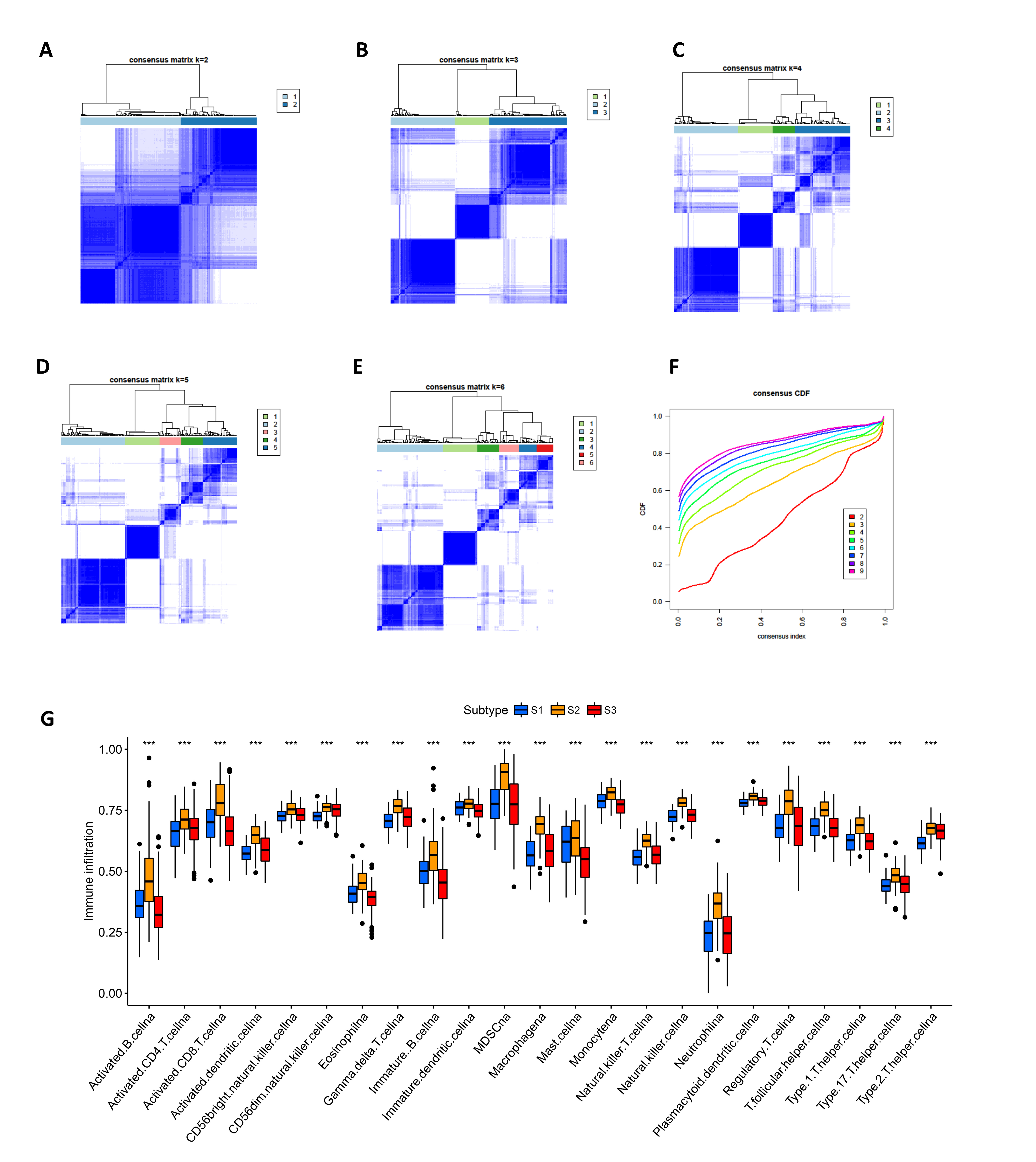

Supplement: Supplementary file 1 [file Image3.JPEG]

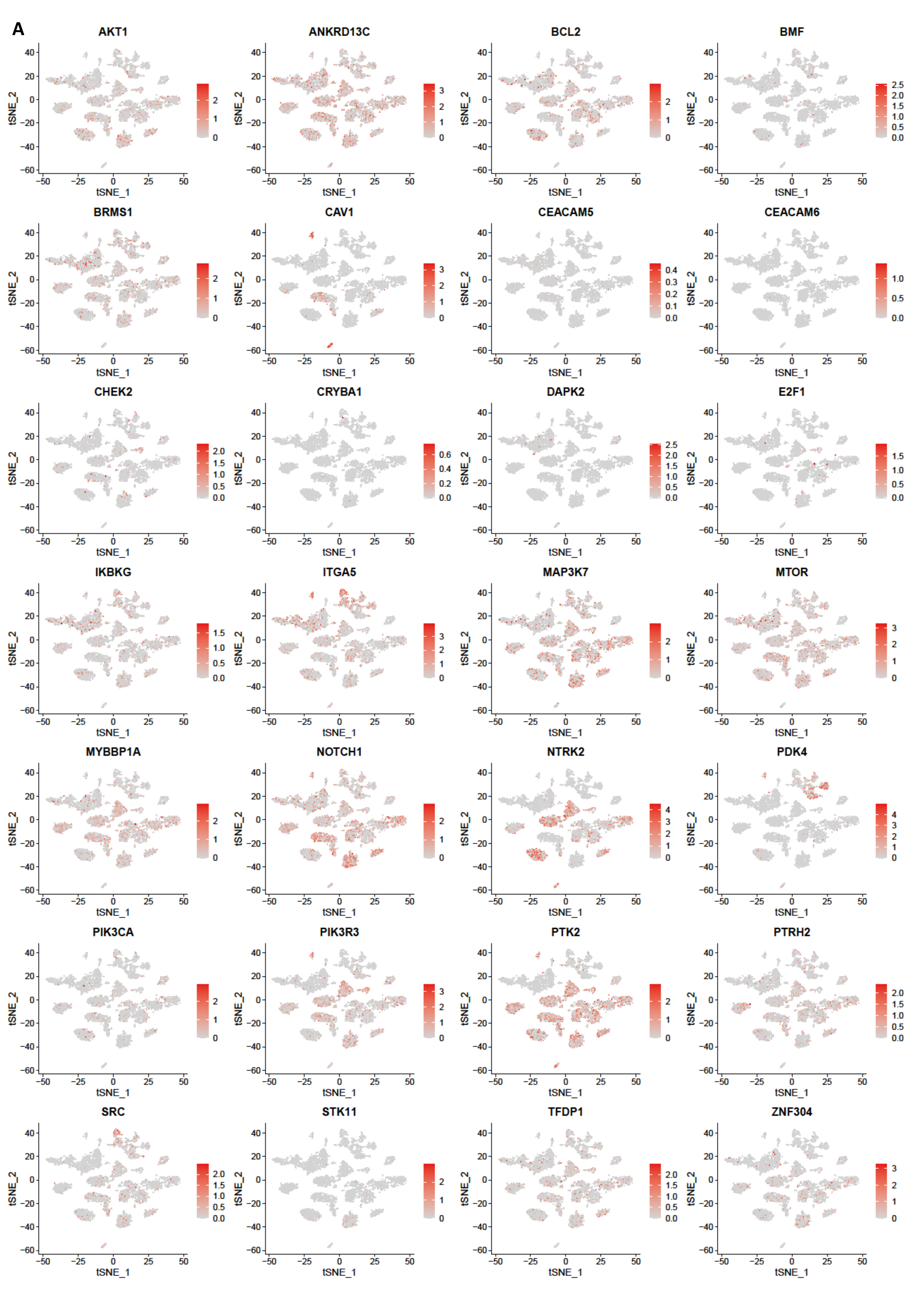

Supplement: Supplementary file 2 [file Image1.JPEG]

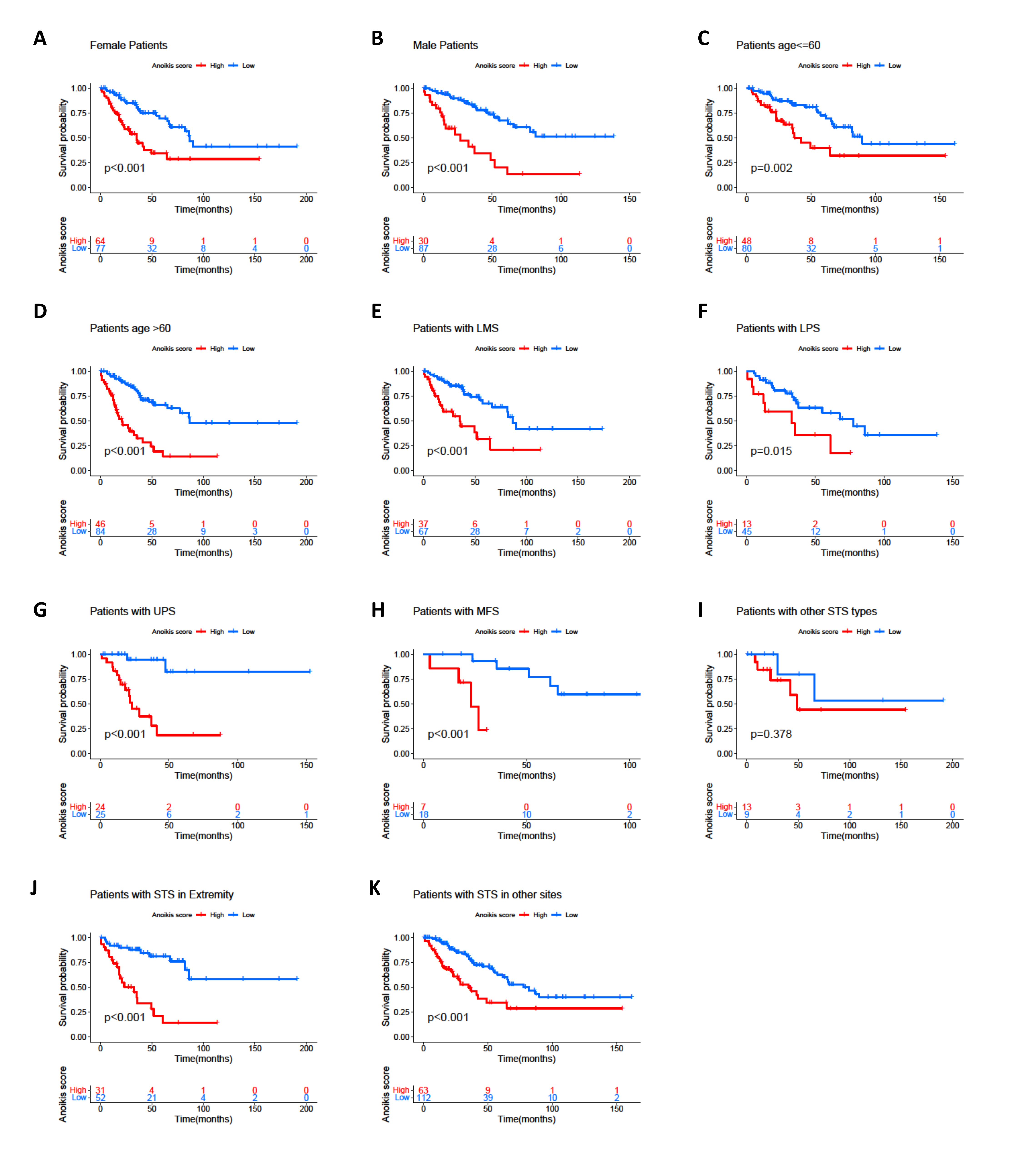

Supplement: Supplementary file 3 [file Image4.JPEG]

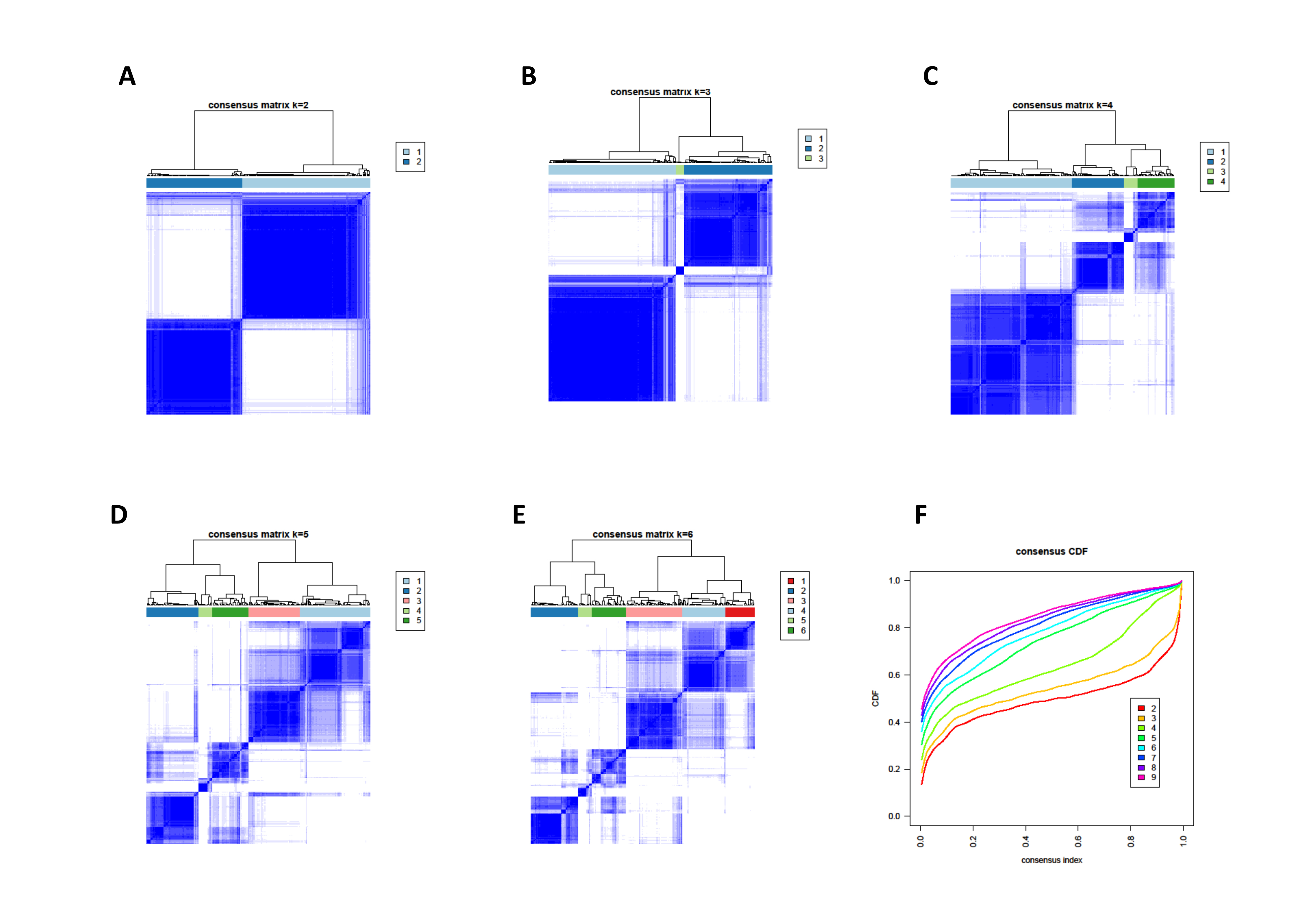

Supplement: Supplementary file 4 [file Image2.JPEG]
